# Supplementary material for: Structural and Functional Outcomes in Rheumatoid Arthritis After 10-Year Therapy with Disease-Modifying Antirheumatic Drugs Under Tight Control: Evidence from Real-World Cohort Data
Source: J Clin Med. 2025 Sep 26;14(19):6832. doi: 10.3390/jcm14196832 (PMC12524530; doi:10.3390/jcm14196832)
Supplement: Supplementary file 1 [file jcm-14-06832-s001.zip › Supplementary Table S2.pdf]

Table S2. Predictors of physical remission after 10-year DMARD therapy in RA patients who had poor or moderate control of clinical disease activity

Variables at baseline	Univariable analysis: Unadjusted ORs (95% CI)	Univariable analysis: *p**	Multivariable analysis (Model 1): Adjusted ORs (95% CIs)	Multivariable analysis (Model 1): *p**	Multivariable analysis (Model 2): Adjusted ORs (95% CIs)	Multivariable analysis (Model 2): *p**
Age per additional year	0.96 (0.92–1.01)	0.09	–	–	–	–
Age >65 years, yes vs. no	0.39 (0.15–1.01)	0.052	0.33 (0.11–0.98)	0.047	0.32 (0.10–1.01)	0.32
Male vs. female	2.58 (0.72–9.22)	0.14	–	–	–	–
Anti-CCP, positive vs. negative	0.26 (0.03–2.07)	0.21	–	–	–	–
RF, positive vs. negative	0.33 (0.07–1.48)	0.15	–	–	–	–
RF >200 U/ml, yes or no	1.20 (0.38–3.83)	0.76	–	–	–	–
DAS28-ESR per additional unit	0.48 (0.31–0.72)	<0.001	0.47 (0.30–0.74)	0.001	0.52 (0.33–0.83)	0.005
High (>5.1), yes vs. no	0.29 (0.11–0.73)	0.008	–	–	–	–
Disease duration per additional day	1.00 (1.00–1.00)	0.46	–	–	–	–
≤6 months, yes vs. no	1.59 (0.65–3.93)	0.31	–	–	–	–
Year of the first visit						
2001–2008	1 (reference)	–	–	–	–	–
2009–2011	1.24 (0.45–3.42)	0.67	–	–	–	–
2012–2014	1.62 (0.50–5.29)	0.42	–	–	–	–
Previous use of cs DMARDs, yes vs. no	0.77 (0.24–2.53)	0.67	–	–	–	–
Smoking history >30 pack-years, yes vs. no	2.28 (0.50–10.41)	0.29	–	–	–	–
BMI >25, yes vs. no	0.05 (0.01–0.24)	<0.001	0.54 (0.16–1.83)	0.32	0.59 (0.16–2.12)	0.42
mTSS						

0 (no erosion/normal joint space)	1 (reference)	–	1 (reference)	–	–	–
>0 and ≤5	0.23 (0.068–0.84)	0.030	0.13 (0.02–0.72)	0.02	–	–
>5 and ≤10	0.16 (0.04–0.60)	0.007	0.11 (0.02–0.59)	0.01	–	–
>10	0.08 (0.02–0.32)	<0.001	0.08 (0.01–0.77)	0.03	–	–
Erosion score						
0 (no erosion)	1 (reference)	–	–		1 (reference)	–
>0 and ≤3	0.31 (0.10–0.89)	0.03	–		0.52 (0.15–1.77)	0.29
>3 and ≤10	0.33 (0.08–1.32)	0.12	–		1.32 (0.20–8.81)	0.78
>10	0.35 (0.06–2.05)	0.25	–		0.79 (0.09–6.84)	0.83
JSN score						
0 (normal joint space)	1 (reference)	–	–		1 (reference)	–
>0 and ≤3	0.28 (0.08–1.07)	0.064	–		0.31 (0.07–1.34)	0.12
>3 and ≤10	0.34 (0.08–1.38)	0.13	–		0.26 (0.05–1.24)	0.09
>10	0.11 (0.03–0.36)	<0.001	–		0.12 (0.02–0.58)	0.009

*Univariable and multivariable logistic regression analyses were conducted to evaluate baseline characteristics associated with physical remission after 10 years of DMARD therapy under tight control. Physical remission was defined as HAQ-DI after 10 years ≤0.5. All variables with *p*-values <0.10 in the univariable models were introduced into multivariable analysis as independent factors using a forced entry procedure. As independent factors, mTSS was introduced into Model 1, and erosion score and JSN score were introduced into Model 2. The multivariable model yielded an AUC–ROC of 0.81 (0.72–0.90, *p* <0.001) for Model 1 and 0.82 (0.72–0.91, *p* <0.001) for Model 2.

anti-CCP, anti-cyclic citrullinated peptide antibodies; AUC, area under the curve; BMI, body mass index; csDMARDs, conventional synthetic DMARDs; DAS28-ESR, 28-joint disease activity score using erythrocyte sedimentation rate; DMARD, disease-modifying antirheumatic drug; HAQ-DI, health assessment questionnaire-disability index; mTSS, van der Heijde-modified total Sharp score; ORs, odds ratios; RA, rheumatoid arthritis; RF, rheumatoid factor; ROC, receiver operating characteristic; 95% CIs, 95% confidence intervals.
